# Protective Effects and Mechanism of a Novel Probiotic Strain *Ligilactobacillus salivarius* YL20 against *Cronobacter sakazakii*-Induced Necrotizing Enterocolitis In Vitro and In Vivo

**DOI:** 10.3390/nu14183827

**Published:** 2022-09-16

**Authors:** Weiming Wang, Meng Geng, Caixia Zhu, Lei Huang, Yue Zhang, Tengxun Zhang, Chongjie Zhao, Tongcun Zhang, Xinjun Du, Nan Wang

**Affiliations:** 1 School of Traditional Chinese Medicine, Southern Medical University, Guangzhou 510515, China; 2 Key Laboratory of Industrial Fermentation Microbiology, Ministry of Education and Tianjin, College of Biotechnology, Tianjin University of Science and Technology, Tianjin 300457, China; 3 Tianjin Engineering Research Center of Microbial Metabolism and Fermentation Process Control, Tianjin 300457, China; 4 College of Biotechnology, Tianjin University of Science and Technology, Tianjin 300457, China

**Keywords:** *Cronobacter sakazakii*, intestinal development, *Ligilactobacillus salivarius*, necrotizing enterocolitis, suckling mouse

## Abstract

Exposure to probiotics in early life contributes to host intestinal development and prevention of necrotizing enterocolitis (NEC). *Cronobacter sakazakii* (*C. sakazakii*), an opportunistic pathogen, can cause NEC, bacteremia, and meningitis in neonates, but the research of probiotics against *C. sakazakii* is limited relative to other enteropathogens. Here, the protective effect and mechanism of a novel probiotic *Ligilactobacillus salivarius* (*L. salivarius*) YL20 isolated from breast milk on *C. sakazakii*-induced intestinal injury were explored by using two in vitro models, including an *C. sakazakii*-infected intestinal organoid model and intestinal barrier model, as well as an in vivo experimental animal model. Our results revealed that *L. salivarius* YL20 could promote epithelial cell proliferation in intestinal organoids, rescue budding-impaired organoids, prevent the decrease of mRNA levels of leucine-rich repeat containing G protein-coupled receptor 5 (*Lgr5*), zonula occludens-1 (*Zo-1*) and *Occludin*, and reverse *C. sakazakii*-induced low level of Mucin 2 (MUC2) in intestinal organoids. Additionally, YL20 could inhibit *C. sakazakii* invasion, increase the expression of ZO-1 and occludin in *C. sakazakii*-infected HT-29 cells, and reverse TEER decrease and corresponding permeability increase across *C. sakazakii*-infected Caco-2 monolayers. Furthermore, YL20 administration could alleviate NEC in *C. sakazakii*-infected neonatal mice by increasing the mice survival ratio, decreasing pathology scores, and downregulating pro-inflammatory cytokines. Meanwhile, YL20 could also enhance intestinal barrier function in vivo by increasing the number of goblet cells, the level of *MUC-2* and the expression of ZO-1. Our overall findings demonstrated for the first time the beneficial effects of *L. salivarius* YL20 against *C. sakazakii*-induced NEC by improving intestinal stem cell function and enhancing intestinal barrier integrity.

## 1. Introduction

Necrotizing enterocolitis (NEC), one of the most devastating gastrointestinal diseases with high morbidity and mortality, mainly occurs in newborn premature infants and exhibits an incidence of 15~20% in premature infants with a weight <1000 g [1,2], an average mortality of 15–30%, and even as high as 75–85% in its most severe forms [3,4]. The exact pathogenesis of NEC has been an area of intense investigation, and several risk factors, including prematurity, formula feeding, and the presence of pathogens, may cause NEC by stimulating intestinal inflammatory response, destroying enteric mucosal integrity, and inducing intestinal epithelial necrosis [5,6]. Notably, *Cronobacter sakazakii* (*C. sakazakii*), one of opportunistic pathogens, is implicated in severe outbreaks of NEC in premature and full-term infants, suggesting its potential important role in the pathogenesis of NEC [7]. *C. sakazakii* is a Gram-negative, facultative anaerobic, peritrichously flagellated, rod-shaped, and non-spore-forming pathogen in the *Enterobacteriaceae* family. It is mainly present in many low moisture foods like infant formula, and also in hospitals, homes, and equipment such as enteral feeding tubes and baby bottles. *C. sakazakii* can cause severe NEC, neonatal meningitis, and bacteremia, with mortality rates ranging from 40% to 80% for premature (<37 weeks of gestational age) and/or low-birth weight infants (<2500 g) [8,9]. Despite clinical recovery with antibiotics, the survivors of *C. sakazakii* infection are often accompanied by chronic sequelae, such as neurological impairment and developmental disorders [10].

Currently, probiotics have received significant attention due to their potential in the prevention and treatment of NEC [11]. Administering probiotics at the early stage can effectively improve intestinal health, promote intestinal development, and relieve intestinal damage by promoting intestinal motility and absorption, enhancing intestinal barrier integrity, stimulating immune system maturation, and balancing intestinal microbiota. Probiotics may resist pathogens through direct antagonism (competition for nutrition or epithelial adhesion sites), or indirect antagonism (release of metabolites such as organic acids, etc.). Clinical studies have confirmed the beneficial effects of probiotics including *Bifidobacterium longum subsp. infantis* (previously known as *Bifidobacteria infantis*) (*B.* *infantis*) and *Lactobacillus acidophilus* on NEC [12,13]. Several probiotic strains, including *Bacteroides fragilis* ZY-312 [5] and *Lactococcus lactis* [14], have also been reported to protect against *C. sakazakii*-induced intestinal inflammation in two different models, a neonatal rat model and a preterm rabbit model. A conditioned medium from *B. infantis* was found to be able to prevent *C. sakazakii*-induced NEC in newborn mice [15]. However, the research of probiotics against *C. sakazakii* is limited relative to other enteropathogens.

Infectious intestinal pathogens, including *C. sakazakii*, induced NEC through adhering to intestinal epithelial cells and damaging the intestinal barrier. Intestinal stem cell (ISC)-driven renewal of epithelial cells in crypts help maintain the intestinal barrier integrity against pathogen invasion, repair the damaged site, and prevent intestinal inflammation [16]. Studies on germ-free animals have shown that colonized intestinal microorganisms can influence early intestinal morphological development, intestinal epithelial cell proliferation and differentiation [17]. Intestinal organoids containing ISCs can generate all the differentiated lineages, including absorptive cells, goblet cells, enteroendocrine cells, paneth cells, etc., indicating that the intestinal organoid is an excellent model in studying host–microbial interactions in vitro [18]. *Ligilactobacillus* (formerly known as *Lactobacillus*) *salivarius* (*L. salivarius*) is generally considered as a beneficial probiotic and widely used in dairy foods and nutritional supplements. However, whether *L. salivarius* can inhibit *C. sakazakii*-induced NEC has been unknown.

To explore the protective effects and related mechanisms of our newly identified probiotic strain (*L. salivarius* YL20) on *C. sakazakii*-induced NEC, we first compared the stimulatory effects on intestinal epithelial development using the in vitro model of intestinal organoids and antagonistic activity against *C. sakazakii* between *L. salivarius* YL20 and other probiotic strains (*Lactobacillus gasseri* GM20, *Lacticaseibacillus rhamnosus* SW01, *Lacticaseibacillus rhamnosus* GG (LGG), etc.). The beneficial effects of *L. salivarius* YL20 on *C. sakazakii*-infected intestinal epithelial proliferation and repair were further investigated by using a caco-2 monolayer model and an experimental mice model. Our results suggest that *L. salivarius* YL20 could protect against *C. sakazakii*-induced intestinal inflammation by improving intestinal stem cell function and enhancing intestinal barrier.

## 2. Materials and Methods

### 2.1. Bacterial Strains and Cell Culture

*L. salivarius* YL20 isolated from the breast milk of healthy mothers was confirmed by 16S RNA sequencing (GenBank: MT597593.1). *C. sakazakii* strain (ATCC No. 51329) was grown in lysogeny broth (LB) medium and all lactobacillus strains (*Lacticaseibacillus salivarius* YL20, *Lactobacillus gasseri* GM20, *Lacticaseibacillus rhamnosus* SW01, *Lacticaseibacillus paracasei* TX01, *Lactobacillus delbrueckii* Y2 and *Streptococcus thermophilus* ZY9) were cultured in MRS broth at 37 °C. HT-29 and Caco-2 cells were cultured in DMEM medium with 10% FBS, 100 mg/mL streptomycin, and 50 mg/mL penicillin G.

### 2.2. Intestinal Organoid Culture

Organoids from small intestines of C57BL/6 mice (4-week-old) were isolated and cultured as reported by Lee et al. [19]. After mouse intestines were cut into pieces, a gentle cell dissociation reagent (Stemcell, Vancouver, BC, Canada) was used to isolate intestinal crypts, and digestion was terminated by adding 0.1% bovine serum albumin (BSA) in PBS. Next, the intestinal crypt extract was filtrated by using a 70 μm cell strainer, centrifuged at 290× *g* for 5 min, followed by resuspending the pellets in complete medium (Stemcell, Vancouver, BC, Canada). After mixing the suspension with Matrigel at a volume ratio of 1:1.5, 50 μL mixture was placed on a pre-warmed 48-well plate, followed by incubation at 37 °C for 10 min, then adding 200 μL of complete medium to each well. The organoids were passaged by a combination of digestion and mechanical disruption.

Intestinal organoids were co-cultured with probiotics as previously described [20]. Briefly, after seeding at 50 organoids per well in a 48-well plate, intestinal organoids were co-incubated with YL20 (10^4^ CFU) for 3 days, and their growth status and morphology were observed under a light microscope. EdU staining, RT-PCR and ELISA were performed as described below.

### 2.3. EdU Staining

The growth of intestinal organoids was detected using an EdU staining kit (Meilunbio, Dalian, China) as instructed by the manufacturer. Briefly, intestinal organoids in each well were incubated with 200 μL of 50 μM EdU medium for 2 h, followed by fixing with paraformaldehyde, permeabilization with 0.5% Triton X-100, and incubation with 1× Apollo reaction cocktail. After staining nuclei with Hoechst 33342, organoids were captured under a fluorescence microscope (Nikon, Tokyo, Japan), and the number of EdU^+^ cells was calculated.

### 2.4. Quantitative Real-Time PCR (qRT-PCR)

Before qRT-PCR analysis, organoids treated with CS or/and YL20 were collected and tissue samples were harvested from mouse small intestines, followed by total RNA extraction using Trizol reagent (Invitrogen, Carlsbad, CA, USA) and RNA reverse transcription using M-MLV reverse transcriptase (Promega, Madison, WI, USA) with the primers listed in Table 1. qRT-PCR was performed with Fast SYBR Green Master Mix using the Biosystems StepOne^TM^ Real-time PCR system (Applied Biosystem, Waltham, MA, USA). The relative amounts of mRNA were analyzed using the 2^−ΔΔCT^ method.

### 2.5. Enzyme Linked Immunosorbent Assay (ELISA)

The level of Mucin 2 (MUC2) in the organoids was detected by co-culturing intestinal organoids with probiotics, followed by collecting the supernatant for ELISA. To detect the levels of MUC2 and cytokines in the small intestines, small intestines were collected from euthanized mice, followed by homogenization, centrifugation, and measuring MUC2 (Cusabio, Wuhan, China), IL-1β (Solarbio, Beijing, China), IL-6 (Solarbio, Beijing, China), and TNF-α (Solarbio, Beijing, China) using ELISA kits as directed by the manufacturers.

### 2.6. Antagonistic Activity of Probiotics against C. sakazakii Using Agar Well Diffusion Method

The antagonistic activity of probiotics, including YL20, GM20, SW01, and LGG, against *C. sakazakii* was tested using agar well assay as previously reported with a few modifications [21]. Briefly, the overnight grown probiotic strains in MRS broth were centrifuged (2000× *g* for 5 min) to obtain supernatants. Meanwhile, the overnight (12 h) cultures of *C. sakazakii* were laid on the LB agar surface, followed by covering the surface with an Oxford cup (outer diameter 7.8 ± 0.1 mm, inner diameter 6.0 ± 0.1 mm, and height 10.0 ± 0.1 mm) containing 200 μL of each probiotic supernatant. After 24 h of incubation, the inhibition zone diameter (mm) was measured. The test was performed in triplicate and repeated three times.

### 2.7. Sensitivity to Antibiotics

The detection of YL20 susceptibility to antibiotics was performed followed a previously reported disk-diffusion method [22]. Briefly, the strain was cultured in MRS broth at 37 °C overnight and then diluted in MRS liquid medium to a final inoculation concentration of 10^7^ CFU/mL. Next, 100 µL of diluted culture was coated onto MRS agar, and the antibiotic discs were put on agar surface. After incubation at 37 °C overnight, inhibition zone diameters were measured using a digital caliper. The test was performed in triplicate and repeated three times.

### 2.8. Invasion Assay

**Figure 1:**
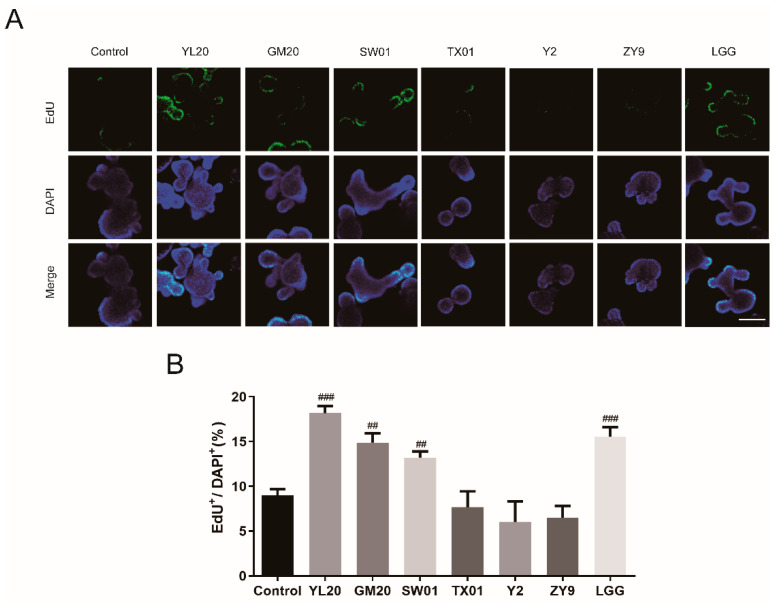
Effects of lactic acid bacteria on intestinal organoid proliferation. (**A**) Images of intestinal organoids stained with Hoechst33342 (blue) and EdU (green); scale bar = 50 μm. (**B**) Percentage of EdU-positive cells calculated for different lactic acid bacteria. ## *p* < 0.01, ### *p* < 0.001 vs. Control group, *n* = 3.

The inhibitory effects of YL20 against *C. sakazakii* adhering to HT29 cells were investigated by competition, exclusion, and replacement assays according to Fan et al. [5]. Briefly, after seeding at 1 × 10^5^ cells per well in a 24-well plate, HT-29 cells were incubated with 1 × 10^7^ CFU of *C. sakazakii* for 3 h (Control group). In a competition assay, the cells were incubated with both 1 × 10^8^ CFU of YL20 and 1 × 10^7^ CFU of *C. Sakazakii* for 3 h. To detect the replacement of *C. sakazakii* by YL20, cells were infected with 1 × 10^7^ CFU of *C. sakazakii* for 1.5 h, followed by treatment with 1 × 10^8^ CFU of YL20 for additional 1.5 h. In an exclusion assay, cells were preincubated with 1 × 10^8^ CFU of YL20 for 1.5 h, followed by infection with 1 × 10^7^ CFU of *C. Sakazakii* for additional 1.5 h (Figure 1A). After washing with PBS to remove the unbound bacterial cells, the HT-29 cells were treated with 0.1% Triton X-100 for 10 min, followed by culturing the broken cells with *C. sakazakii* on the LB agar plates and then calculating the number of intracellular *C. sakazakii* cells. The results were presented as the percentage of the invasive bacteria (% CFU invasive bacteria in experiment group/CFU invasive bacteria in control group).

### 2.9. Western Blotting Analysis

Briefly, after lysis with RIPA lysis buffer, the protein concentrations of HT-29 cells were analyzed using a BCA protein quantitative reagent kit (Solarbio, Beijing, China). After sodium dodecyl sulphate-poly acrylamide gel electrophoresis (SDS-PAGE), the extracted proteins were transferred to a nitrocellulose membrane, probed with primary antibodies against ZO-1 (21773-1-AP, Proteintech Group, Inc., Wuhan, China), occludin (sc-133255, Santa Cruz Biotechnology, Inc., Santa Cruz, CA, USA) and GAPDH (YM3215, ImmunoWay Biotech., Plano, TX, USA) overnight at 4 °C, followed by incubation with secondary antibodies IRDye-800CW IgG (926-32210, Li-COR Biosciences, Lincoln, NE, USA) or IRDye-680RD IgG (926-68071, Li-COR Biosciences, Lincoln, NE, USA) for 2 h at room temperature. The specific bands were analyzed using an Odyssey Infrared Imaging System (Li-COR Biosciences) and quantified using ImageJ software.

### 2.10. Immunofluorescence Analysis

After incubation with bacteria, Caco-2 cells were fixed in 4% paraformaldehyde for 30 min at room temperature and permeabilized with 0.5% Triton X-100 for 5 min. Subsequently, the cells were blocked in 5% BSA for 1 h at room temperature, followed by immunostaining with anti-ZO-1 antibody (21773-1-AP, Proteintech Group, Inc., Wuhan, China) and tetramethyl rhodamine isothiocyanate (TRITC)-coupled antibody (ZF-0316, ZSGB-BIO, Beijing, China). After nuclei staining with 4′,6-diamidino-2-phenylindole (DAPI), the cells were observed and photographed under a fluorescence microscope.

### 2.11. Measurement of Transepithelial Electrical Resistance (TEER) and Permeability across Caco-2 Monolayers

After seeding in transwell inserts, Caco-2 cells were cultured for at least 16 days to form monolayers. For TEER measurement, the monolayers were incubated with 1 × 10^8^ CFU of YL20 and 1 × 10^7^ CFU of *C. sakazakii*, followed by measuring TEER at 0, 6, 18 and 24 h after bacterial exposure using a Millicell electrical resistance apparatus (World Precision Instruments Inc., Sarasota, FL, USA). For paracellular permeability assays, the chambers were equilibrated with Hanks, followed by adding FITC-dextran (1 mg/mL) (Sigma, St Louis, MO, USA) to the upper chamber and incubation at 37 °C for 1.5 h in the dark. Next, the solutions in the lower chamber were collected, followed by measuring the fluorescence intensity at 520 nM with a microplate reader (Tecan, Zürich, Switzerland). As previously reported [23], apparent permeability coefficient (Papp) was counted by the formula: Papp = d*Q*/(d*t* × *A* × *C*_0_), where d*Q*/d*t* is the permeability rate; *A*, the diffusion area of monolayer; and *C*_0_, the initial concentration of the tested substance in the apical compartment.

### 2.12. Animal Experiment

All procedures were approved by the Animal Ethics Committee of Tianjin University of Science and Technology. Specifically, pregnant C57BL/6 mice were obtained from the PLA Military Academy of Medical Sciences Laboratory Animal Center (Beijing, China), and after delivery, the newborn pups were kept with their mothers and divided into four groups (7 mice/per goup): Control (PBS-treatment), CS (*C. sakazakii*-treatment), YL20 (YL20 treatment), and CS + YL20 (co-treatment with *C. sakazakii* and YL20). Specifically, two-day-old pups were gavaged once per day with 1 × 10^7^ *C. sakazakii* (CS) or 1 × 10^7^ YL20 (YL20) or both (CS + YL20) in 10 μL sterile PBS, with 10 μL PBS fed once per day for the Control pups. Body weight was recorded every day. After 10 days of experiment, all pups were euthanized, and the entire small intestines were collected, with one part of intestinal tissues fixed with 4% paraformaldehyde, and the other part directly frozen in liquid nitrogen and stored at −80 °C.

### 2.13. H&E Staining, PAS Staining, and Immunohistochemical Staining

Small intestine segments fixed in paraformaldehyde solution were paraffin embedded and then cut into 5 µM sections, followed by serial dehydration in xylene and ethanol. For observing intestine architecture, the sections were stained with hematoxylin and eosin (H&E). Pathology/inflammation scores was determined based on a previous report [5]. Goblet cells were detected by periodic acid-Schiff (PAS) staining using an AB-PAS Stain Kit (Solarbio, Beijing, China) as guided by the manufacturer. For ZO-1 staining, the sections were blocked in 1% normal goat serum and incubated overnight at 4 °C with an anti-ZO-1 antibody (21773-1-AP, Proteintech Group, Inc., Wuhan, China), and then for 1 h at room temperature with a TRITC-coupled secondary antibody (ZF-0316, ZSGB-BIO, Beijing, China). After nucleus staining with DAPI, all sections were observed and captured using a forward fluorescence microscope (Olympus, Tokyo, Japan).

### 2.14. Quantification of the Predominant Bacterial Groups in the Intestinal Contents

The genomic DNA of colonic and cecal contents were extracted using the stool genomic DNA extraction kit (Solarbio, Beijing, China). qRT-PCR was performed with Fast SYBR Green Master Mix using the Biosystems StepOne^TM^ Real-time PCR system using primers that specifically target 16S rDNA (Table 2). The amplification was programed to start at 95 °C for 2 min, followed by 40 cycles (degeneration at 95 °C for 10 s, annealing at 60 °C for 30 s). Overall, 16 S rRNA gene abundance in samples was determined by comparing the cycle threshold (Ct) values to those generated by a standard curve.

### 2.15. Statistical Analysis

GraphPad Prism9 (GraphPad Software, San Diego, CA, USA) was used to assess statistical differences between experimental groups. Statistical differences between two experimental groups were evaluated by Student’s *t* tests, and statistical differences between multiple groups were calculated using one-way ANOVA followed by Tukey’s post hoc test. Shapiro–Wilk test was used to analyze the normality of variance. The homogeneity test of variance was performed by running one-way ANOVA analysis. Brown–Forsythe and Welch ANOVA test was performed when the variances are not equal. A *p*-value of 0.05 or lower was considered statistical significant and expressed as * *p* < 0.05, ** *p* < 0.01, and *** *p* < 0.001.

## 3. Results

### 3.1. Effects of Lactic Acid Bacteria on Intestinal Organoid Proliferation

To assess the stimulatory effect of lactic acid bacteria on intestinal epithelial cells, intestinal organoids were isolated from mice small intestines as an in vitro model for studying host–microbial interactions. A total of six strains, including *Lacticaseibacillus salivarius* YL20, *Lactobacillus gasseri* GM20, *Lacticaseibacillus rhamnosus* SW01, *Lacticaseibacillus paracasei* TX01, *Lactobacillus delbrueckii* Y2 and *Streptococcus thermophilus* ZY9 were isolated from breast milk and newborn feces and used for preliminary evaluation, with LGG as a positive control strain. As shown in Figure 1A,B, compared with control group, YL20, GM20 and SW01 could significantly promote cellular proliferation in organoids (*p* < 0.01, *p* < 0.001), with an EdU positive cell percentage of 18.17 ± 0.80%, 14.89 ± 1.02% and 13.22 ± 0.69%, respectively. Meanwhile, the control strain LGG showed an EdU positive cell percentage of 15.53 ± 1.08%. Additionally, the resistance of these three strains and LGG to *C. sakazakii* was detected by agar well diffusion method. In Table 3, it was shown that, compared with the other lactic acid bacteria, YL20 showed a larger bacteriostatic circle against *C. sakazakii*. In Table 4, antibiotic susceptibility test results indicated that YL20 was resistant to penicillin and streptomycin, but susceptible to other antibiotics, indicating that *L. salivarius* YL20 could be used for subsequent experiments.

### 3.2. L. salivarius YL20 Attenuates C. sakazakii-Induced Damage of Intestinal Organoids by Promoting ISC-Mediated Epithelial Cell Proliferation and Enhancing Intestinal Barrier Function

**Figure 2:**
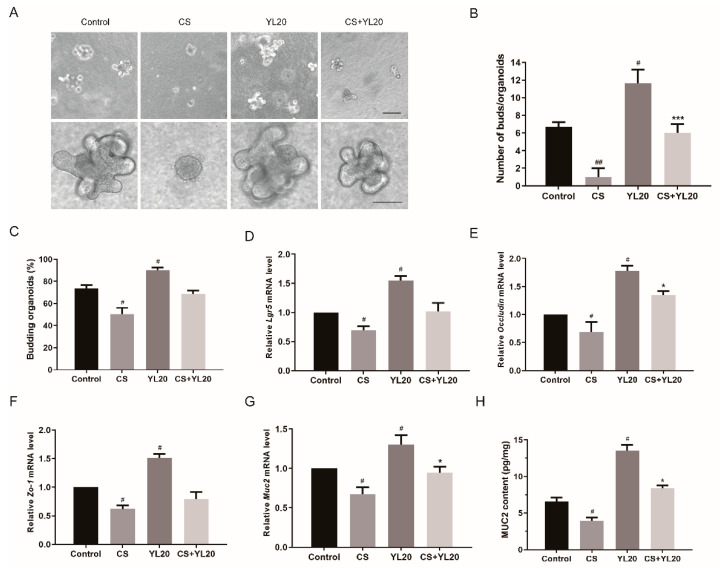
Effects of *L. salivarius* YL20 on *C. sakazakii*-induced damage of intestinal organoids. (**A**) Effects of YL20 on the growth of *C. sakazakii*-infected organoids; the upper four images scale bar = 50 μm, the lower four images scale bar = 100 μm. (**B**) The number of buds per organoid. (**C**) The percentage of budding organoids. Effects of YL20 on the mRNA level of *Lgr5* (**D**), *Occludin* (**E**), *Zo-1* (**F**) and *Muc2* (**G**) in *C. sakazakii*-infected organoids tested by RT-PCR. (**H**) The expression of MUC2 in the supernatant of intestinal organoids. # *p* < 0.05, ## *p* < 0.01 vs. Control group; * *p* < 0.05; *** *p* < 0.001 vs. CS group; *n* = 3.

The isolated small intestinal crypts formed bud structures and protruded outward for 2 to 3 d. The intestinal organoids were co-treated with YL20 and *C. sakazakii* for 2 d. As shown in Figure 2A–C, compared with the Control group, YL20 group showed a significant increase in the volume of organoids, and a notable increase in the number of buds and budding rate of organoids (*p* < 0.05). Furthermore, *C. sakazakii* infection caused the disappearance of buds and the disruption of organoids (*p* < 0.05, *p* < 0.01). Compared with the CS group, the CS + YL20 group could significantly inhibit the *C. sakazakii*-induced damage of organoids, with a significant increase in bud number (*p* < 0.001). Additionally, compared with the Control group, the CS group showed a decrease in the mRNA level of intestinal stem cell marker *Lgr5* (*p* < 0.05), while the *Lgr5* mRNA level was restored in CS + YL20 group (Figure 2D), suggesting that YL20 could promote intestinal stem cell proliferation. YL20 could also up-regulate the mRNA level of tight junctional protein *Occludin* (*p* < 0.05) and *Zo-1* (*p* < 0.05) in *C. sakazakii*-infected organoids (Figure 2E,F). The *C. sakazakii*-induced decrease in the mRNA and protein level of MUC2 could be partly restored in the CS + YL20 group (*p* < 0.05) (Figure 2G,H), indicating the contribution of YL20 to the improvement of intestinal mucosal barrier. These results confirmed that *L. salivarius* YL20 could alleviate *C. sakazakii*-induced damage of intestinal organoids by increasing ISCs-mediated intestinal epithelial cell proliferation and improving intestinal barrier integrity.

### 3.3. L. salivarius YL20 Inhibits C. sakazakii Adherence to Intestinal Cells and Enhances the Expression of TJ Proteins in Intestinal Epithelial Cells

**Figure 3:**
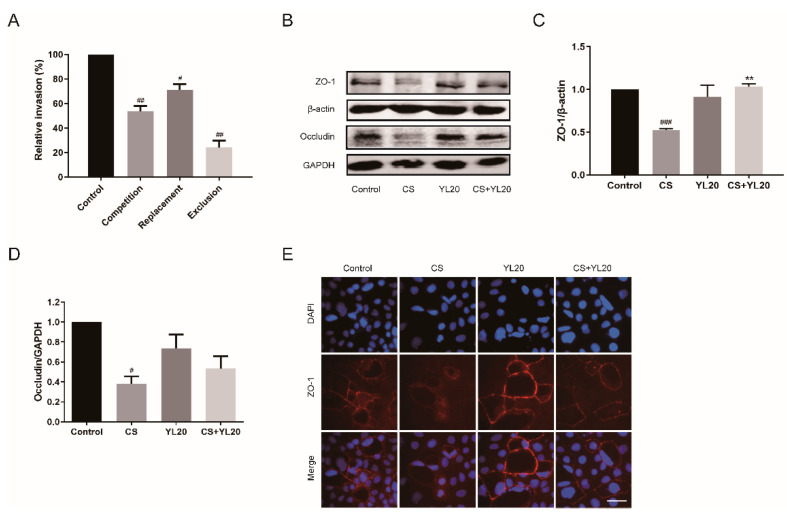
Effects of *L. salivarius* YL20 on *C. sakazakii* adherence to intestinal cells and the expression of TJ proteins in intestinal epithelial cells. (**A**) Effects of YL20 on *C. sakazakii* invasion of HT-29 cells. The results were presented as the percentage of the invasive bacteria (% CFU invasive bacteria in the experiment group/CFU invasive bacteria in the control group). (**B**) Western blotting analysis of the effects of YL20 on the expression of ZO-1 and occludin in *C. sakazakii*-infected HT-29 cells. (**C**,**D**) Quantification of the TJ proteins using Image J software. (**E**) Immunofluorescent staining analysis of ZO-1, scale bar = 50 μm. # *p* < 0.05, ## *p* < 0.01, ### *p* < 0.001 vs. Control group; ** *p* < 0.01 vs. CS group; *n* = 3.

The adhesion and invasion ability of *C. sakazakii* to host intestinal epithelial cells is critical to disease pathogenesis. *C. sakazakii* is considered to invade and translocate into the blood stream to cause infection through intestinal epithelial cells [30], suggesting that the ability of probiotics to prevent/reduce pathogenic bacteria adherence to intestinal epithelial cells is very important in the prevention of pathogen infection. The potential antagonistic properties of YL20 against *C. sakazakii* were explored by using an in vitro model based on HT-29 cells. According to the adding sequence of YL20 and *C. sakazakii* into cell co-cultures, three adhesion experiments (competition, replacement, and exclusion) were designed, and the results are shown in Figure 3A. YL20 was shown to expel *C. sakazakii* from HT-29 cells with almost 70–80% efficiency (*p* < 0.01), suggesting its suppressive effect against *C.* *sakazakii* infection in HT-29 cells. Additionally, the effect of YL20 on epithelial barrier function was further investigated by analyzing tight junction protein expression in the intestine cell lines. In Figure 3B–D, *C. sakazakii* infection was seen to significantly decrease the expression of ZO-1 (*p* < 0.001) and occludin (*p* < 0.05), whereas YL20 pretreatment could significantly increase ZO-1 expression (*p* < 0.01) in *C. sakazakii*-infected HT-29 cells. Meanwhile, YL20 could also increase the occludin expression in *C. sakazakii*-infected HT-29 cells, but not significantly. Immunofluorescence staining confirmed that YL20 pretreatment could prevent the *C. sakazakii*-induced destruction of ZO-1 expression in Caco-2 cells (Figure 3E). These results indicated that YL20 could inhibit *C. sakazakii* adhesion to epithelial cells and *C. sakazakii*-induced low TJ protein expression.

### 3.4. L. salivarius YL20 Reverses TEER Decrease and Corresponding Membrane Permeability Increase across C. sakazakii-Infected Caco-2 Monolayers

**Figure 4:**
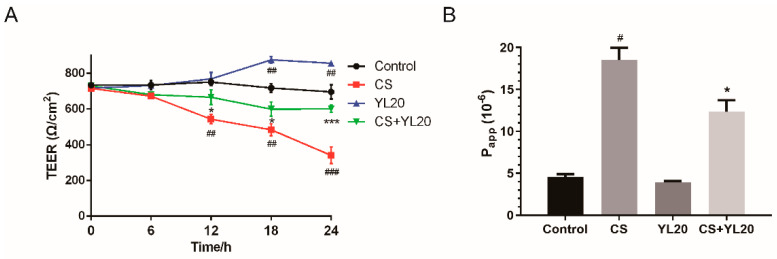
Effects of *L. salivarius* YL20 on TEER and membrane permeability of *C. sakazakii*-infected Caco-2 monolayers. (**A**) During co-incubation of Caco-2 monolayers with YL20 and *C. sakazakii*, TEER was measured at 0, 6, 12, 18, and 24 h. (**B**) Permeability of Caco-2 monolayer to FITC-dextran after treatment with YL20 and/or *C. sakazakii*. # *p* < 0.05, ## *p* < 0.01, ### *p* < 0.001 vs. Control group; * *p* < 0.05, *** *p* < 0.001 vs. CS group; *n* = 3.

The beneficial effect of YL20 on the intestinal barrier function was further tested by co-incubation of caco-2 monolayers with YL20 and *C. sakazakii*, followed by measuring TEER at 0, 6, 12, 18 and 24 h. In Figure 4A, CS treatment was seen to significantly (*p* < 0.001) reduce the TEER of Caco-2 monolayers from 715.67 ± 5.66 Ω/cm^2^ to 340.66 ± 46.65 Ω/cm^2^ in a time-dependent manner, but this TEER reduction was significantly (*p* < 0.001) reversed at 24 h in CS + YL20 treatment group. In Figure 4B, the effect of YL20 on the permeability of Caco-2 monolayers to FITC-dextran was detected following 1.5 h of exposure to *C. sakazakii*. The Papp values were 4.54 × 10^−6^ and 18.51 × 10^−6^ for Control and CS groups, respectively, suggesting that *C. sakazakii* infection could significantly increase permeability (# *p* < 0.05 vs. Control group). Additionally, compared with the CS group, the CS + YL20 group showed a significant (*p* < 0.05) decrease in permeability. These results showed that YL20 could restore TEER, prevent membrane permeability, and enhance intestinal barrier function.

### 3.5. YL20 Administration Attenuates C. sakazakii-Induced Clinical Symptoms and Intestinal Epithelial Damage in Newborn Mice

**Figure 5:**
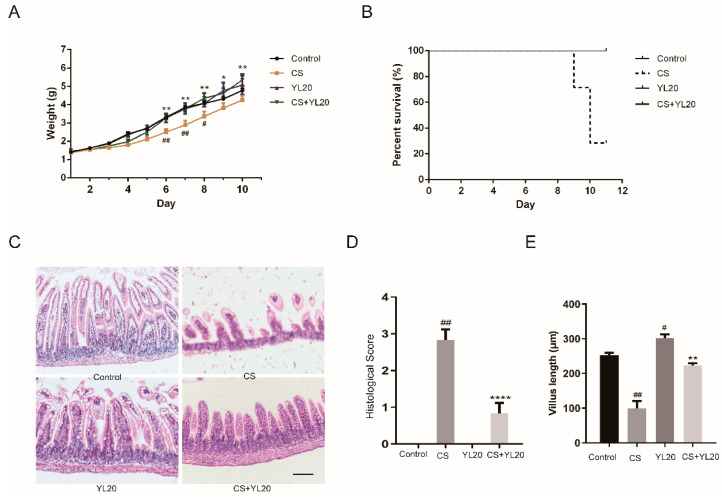
Effects of YL20 on *C. sakazakii*-induced clinical symptoms and intestinal epithelial damage in newborn mice. (**A**) Changes in body weight were monitored daily. (**B**) Mortality rate for neonatal mice in different groups during experiments. (**C**) Images of H&E-stained small intestinal sections from *C. sakazakii*-infected mice with or without YL20 administration; scale bar = 100 μm. (**D**) Pathology/inflammation scores. (**E**) The length of villus was measured in at least 50 villi/mouse. # *p* < 0.05, ## *p* < 0.01 vs. Control group; * *p* < 0.05, ** *p* < 0.01, **** *p* < 0.0001 vs. CS group; *n* = 3–7.

To evaluate whether YL20 could prevent *C. sakazakii*-induced NEC in vivo, newborn mice were gavaged with *C. sakazakii* or YL20 or both once per day from postnatal day 2 (P2) to postnatal day 10 (P10), with 10 μL PBS for Control mice. After infection with the *C. Sakazakii* strain, the mice pups showed severe weight loss from day 6 to 10 (# *p* < 0.05, ## *p* < 0.01 vs. Control group) (Figure 5A). YL20 intervention was shown to be effective in reducing weight loss. Compared with CS pups, CS + YL20 pups showed a significant increase in body weight (* *p* < 0.05, ** *p* < 0.01) (Figure 5A). Meanwhile, the mortality rate of mice was evaluated for each group, and CS pubs began to die on day 9, with a mortality rate of ~70% on day 10, in contrast to no death in the other three groups (Figure 5B).

Furthermore, the intestinal tissues from the *C. sakazakii*-infected mice showed severe epithelial damage, including villus cell loss, epithelial sloughing, and intestinal epithelial cell rupture. In Figure 5D, it was shown that compared with the Control pups, the CS pups showed higher pathological scores (*p* < 0.01), and compared with the CS pups, the CS + YL20 pups showed less intestinal injury (*p* < 0.0001). The morphology of intestines, especially the length of the villus and the ratio of the intestinal villus length to crypt depth, is positively correlated with intestine health [31]. In Figure 5E, the YL20 pups exhibited an increase in villus length relative to the Control pups, suggesting that YL20 could promote intestinal development. Compared with the Control pups, the CS pups showed a decrease of villus length (*p* < 0.01), but this decrease in villus length was significantly reversed in the CS + YL20 pups relative to the CS pups (*p* < 0.01) (Figure 5E). These observations indicated that YL20 could improve mice survival rate and attenuate *C. sakazakii*-induced intestinal injury.

### 3.6. YL20 Administration Inhibits C. sakazakii-Induced Rise of Inflammatory Factors in the Intestinal Tract of Newborn Mice

**Figure 6:**
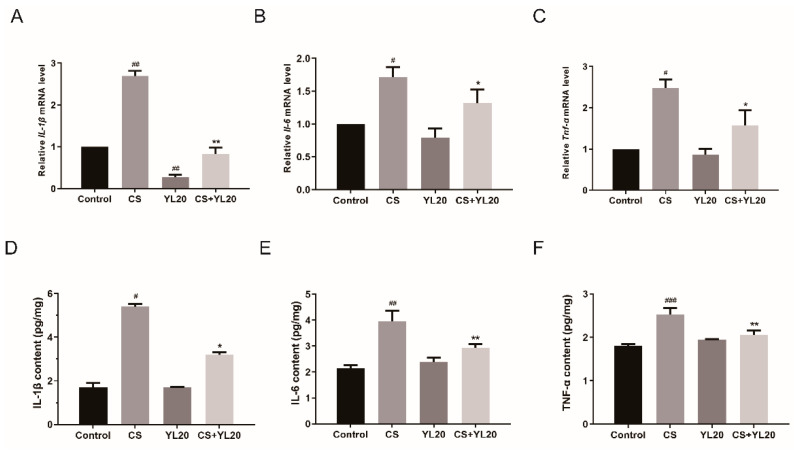
Effects of YL20 administration on *C. sakazakii*-induced inflammatory factors in the intestinal tract of newborn mice. (**A**–**C**) RT-PCR analysis of the mRNA levels of IL-1β, IL-6 and TNF-α in small intestinal tissues from *C. sakazakii*-infected mice with or without YL20 administration. (**D**–**F**) ELISA analysis of the release of pro-inflammatory cytokines. # *p* < 0.05, ## *p* < 0.01, ### *p* < 0.001 vs. Control group; * *p* < 0.05, ** *p* < 0.01 vs. CS group; *n* = 3.

*C. sakazakii*-induced intestinal inflammation is associated with high levels of proinflammatory cytokines. The anti-inflammatory effects of YL20 were investigated by measuring the levels of inflammatory cytokines in mice small intestines with real-time PCR and ELISA. As shown in Figure 6A–C, the CS pups showed significant upregulation in the mRNA levels of IL-1β, IL-6 and TNF-α (*p* < 0.05, *p* < 0.01 vs. Control group), whereas this *C. sakazakii*-induced increase of these proinflammatory cytokines was obviously suppressed in the CS + YL20 pups (*p* < 0.05, *p* < 0.01 vs. CS group). In Figure 6D–F, compared with the Control group, the CS group showed significantly higher levels of these proinflammatory cytokines (*p* < 0.05, *p* < 0.01, *p* < 0.001), but this effect was significantly suppressed in the CS + YL20 group (*p* < 0.05, *p* < 0.01 vs. CS group). These results showed that YL20 could decrease the levels of proinflammatory cytokines, thus contributing to the prevention of *C. sakazakii*-induced NEC.

### 3.7. YL20 Administration Improves Intestinal Barrier Integrity and Inhibits C. sakazakii-Induced Intestinal Barrier Damage

**Figure 7:**
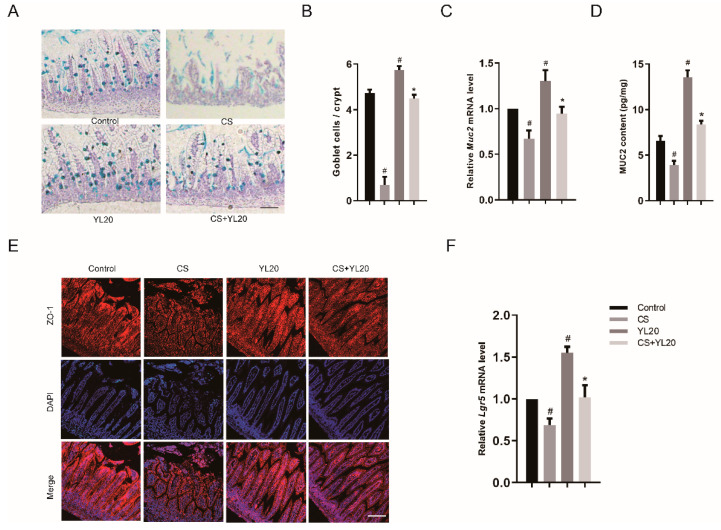
Effects of YL20 administration on intestinal barrier integrity and *C. sakazakii*-induced intestinal barrier damage. (**A**) PAS staining analysis of *C. sakazakii*-infected mice pups with or without YL20 administration; scale bar = 100 μm. (**B**) The number of positively stained cells. (**C**) The mRNA levels of *Muc2* in small intestine tissues of pups by RT-PCR. (**D**) The protein levels of MUC2 in small intestine tissues of pups by ELISA. (**E**) Immunohistochemical staining analysis of ZO-1 expression levels in the intestines of mice pups; scale bar = 100 μm. (**F**) The mRNA levels of *Lgr5* in small intestine tissues of mice pups by RT-PCR. # *p* < 0.05 vs. Control group; * *p* < 0.05 vs. CS group; *n* = 3.

The effects of YL20 on the mucosal barrier in the in vivo model were evaluated by PAS staining analysis of the intestines of mice pups and RT-PCR and ELISA analysis of MUC2 levels. In Figure 7A,B, compared with the Control pups, the CS pups showed a significant decrease in the number of goblet cells (*p* < 0.05), while this reduction was reversed in the CS + YL20 pups relative to the CS pups (*p* < 0.05). In addition, the goblet cell number significantly increased in the YL20 pups relative to the Control pups (*p* < 0.05) (Figure 7B). Moreover, compared with the Control pups, the CS pups showed a notable downregulation in the mRNA level of *Mucin-2* (*p* < 0.05) and this *C. sakazakii*-induced decrease in the mRNA level of MUC2 was obviously suppressed in the CS + YL20 pups (*p* < 0.05 vs. CS group) (Figure 7C). In Figure 7D, the decrease of MUC2 production was observed in the intestines of the CS pups (*p* < 0.05 vs. Control group), but was restored in the CS + YL20 pups (*p* < 0.05 vs. CS group).

The effects of YL20 on TJ protein levels in the in vivo model were analyzed by immunofluorescence staining of ZO-1 in the intestine of mice pups. In Figure 7E, the CS pups were lower than the Control pups in ZO-1 expression. The differences in the response of TJ protein expression to *C. sakazakii* infection with or without YL20 treatment were confirmed by immunoblot analysis of ZO-1, with a higher ZO-1 expression in the YL20 + CS pups than the CS mice pups (Figure 7E).

Furthermore, the effects of YL20 on intestinal stem cell proliferation were determined by RT-PCR analysis of *Lgr5* mRNA level in the intestines of mice pups, and the results are shown in Figure 7F. The mRNA level of *Lgr5* was remarkably increased (*p* < 0.05) in the YL20 pups relative to the Control pups, suggesting that YL20 could promote the proliferation of intestinal stem cells. Meanwhile, the CS pups showed a significant decrease in *Lgr5* mRNA level (*p* < 0.05 vs. Control group), but this *C. Sakazakii*-induced decrease of *Lgr5* mRNA level was effectively curbed in the CS + YL20 pups (*p* < 0.05 vs. CS group). These data supported that YL20 could improve tight junction and intestinal mucosal barrier integrity, thus playing a protective role against *C. sakazakii*-induced NEC.

### 3.8. YL20 Administration Improves Intestinal Microbiota Composition

As shown in Table 5, there was no significant difference in the total bacteria concentrations among the four groups. Compared to the Control group, *C. sakazakii* infection increased the abundance of *Enterobacterium* and *Enterococcus*, and decreased the abundance of *Bifidobacterium*, *Bacteroidetes*, *and Lactobacillus*. However, the administration of YL20 partly restore the proportions of these bacteria, resulting in a higher relative abundance of *Lactobacillus* and a lower relative abundance of *Enterobacterium* in the CS + YL20 group than in the YL20 group. Furthermore, the abundance of *Lactobacillus* was significantly increased and the abundance of *Enterobacterium* and *Enterococcus* was decreased in the YL20 group, compared to the Control group. Both *C. sakazakii* infection and YL20 administration had no significant effect on the populations of *Clostridium perfringens*.

## 4. Discussion

*C. Sakazakii* can cause serious NEC in infants, especially premature infants or low birth weight infants. The mechanism by which this disease affects patients is still not fully understood, thus limiting the development of treatment strategies for *C. sakazakii*-induced NEC other than supportive care. Probiotics have gained increasing attention because of their benefits to human digestive and immune systems. Here, we reported a new probiotic strain *L. salivarius* YL20, which could improve intestinal barrier function and mitigate *C. sakazakii*-induced NEC by promoting intestinal epithelial cell proliferation and repair.

Intestinal epithelial proliferation and replacement is sustained by ISCs, playing a very important role in intestinal barrier maintenance and intestinal injury repair [32]. In previous studies, intestinal organoids cultured in three-dimension (3D) were used as an in vitro model to explore the effects and mechanisms of host–microbial interactions [18]. In this study, we first established an ex vivo model of *C. sakazakii*-infected intestinal organoids and evaluated the effects of *L. salivarius* YL20 on intestinal epithelial proliferation and mucosal barrier function. Our results confirmed that *L. salivarius* YL20 could stimulate epithelial cell proliferation in organoids, promote the growth of intestinal organoids and protect their morphology under *C. sakazakii* infection by increasing the number of buds per organoid, the percentage of budding organoids, and the mRNA level of *Lgr5*. Recently, by using an in vitro intestinal organoid model and in vivo experimental animal model, researchers have investigated the effects of other probiotics on intestinal development as well as ISC growth and differentiation. For instance, neonatal colonization of mice treated with LGG showed enhancement in the proliferation and differentiation of intestinal epithelial cells [33], and the mRNA levels of Toll-like receptor 3 (TLR3) were increased in LGG-treated intestinal organoids [34]. Additionally, *Limosilactobacillus reuteri* D8 was reported to stimulate the growth of intestinal organoids in vitro and ameliorate DSS-induced intestinal mucosa damage in vivo, indicating that this protective effect was achieved through accelerating ISC regeneration [20]. Moreover, by using intestinal organoids and Lgr5^+^ mice, Lee et al. confirmed that *Bifidobacterium* and *Lactobacillus* spp. could stimulate the proliferation of ISCs and the regeneration of intestinal epithelial cells, with lactate as the main effective substance [35]. Consistent with these studies, *L. salivarius* YL20 could activate ISC-mediated intestinal epithelial cell proliferation, thus playing a protective role in *C. sakazakii*-induced intestinal organoid damage.

MUC2, the main component of the mucus layer, is produced by goblet cells and exerts a protective effect against chemical and microbial damage on the intestinal epithelial surface. As reported by Panida Sittipo et al., intestinal organoids derived from irradiated mice showed impaired budding, as well as decreased expression levels of *Lgr5*, *Ki67* and *Muc2*, which could be reversed by heat-killed *Lactobacillus acidophilus* [36]. Several lactobacillus strains were shown to induce mucin expression, thereby blocking adhesion of pathogens to the intestinal epithelial [37,38,39]. For instance, *Lactobacillus* spp. could upregulate MUC3 expression in human intestinal epithelial cells, while stimulating MUC2 production and secretion [38,40]. *Limosilactobacillus reuteri* could attenuate DSS-induced colitis in mice by increasing mucus thickness [41]. Here, YL20 could up-regulate the mRNA level of *Muc2* and stimulate MUC2 secretion in *C. sakazakii*-infected intestinal organoids.

Tight junctions are vital to maintain intestinal barrier integrity. The formation of TJ is very important to prevent the paracellular spread of bacteria, thus contributing to the resistance of intestinal inflammatory and infectious diseases in the infantile period. Several important epithelial TJ proteins, such as ZO-1, occludin, etc., contributed to the maintenance of intestinal epithelial barrier integrity [42]. Here, we investigated whether YL20 could increase the expression of these TJ proteins in intestinal organoids and intestinal epithelial cell lines. Our data indicated that YL20 treatment could reverse the *C. sakazakii*-induced low mRNA levels of *Zo-1* and *Occludin* in intestinal organoids and restore their respective protein levels in *C. sakazakii*-infected HT-29 cells. Immunostaining of ZO-1 also indicated the protective effect of YL20 on the maintenance of tight junction integrity in Caco-2 cells. Our results are consistent with two previous studies on *B. fragilis* ZY-312 and *L. salivarius.* Specifically, pretreatment of *B. fragilis* ZY-312 could increase ZO-1 and occludin expression in *C. sakazakii*-infected Caco-2 cells [5]. *L. salivarius* was shown to increase the levels of ZO-1 and occludin in enterotoxigenic *Escherichia coli* (ETEC) K88 infected IPEC-J2 cells [43].

Adhesion and invasion are critical steps for *C. sakazakii* to enter host cells, leading to intestinal and extraintestinal diseases. Many isolated strains of *C. sakazakii* were reported to have the ability to adhere to human intestinal epithelial and endothelial cells [44], suggesting preventing *C. sakazakii* adhesion to intestinal epithelium could effectively inhibit its infection. Multiple probiotics, including *B. fragilis* ZY-312 [5], *Lacticaseibacillus paracasei* FJ861111.1 [21], etc. [45,46], were found to inhibit the adhesion of *C. sakazakii* to epithelial cells and intestinal mucus. Consistent with these studies, our results demonstrated that YL20 could inhibit the adhesion of *C. sakazakii* to epithelial cells in competition, replacement, and exclusion assays.

Intestinal barrier integrity is very important for resisting the invasion of pathogens. TEER and permeability assays were performed to study the protective effect of YL20 on *C. sakazakii*-induced epithelial barrier damage in Caco-2 monolayers. Our results showed that the TEER of *C. sakazakii*-infected Caco-2 monolayers decreased gradually over time, indicating the disruptive effect of *C. sakazakii* on epithelial barrier. However, YL20 treatment could reverse *C. sakazakii*-induced TEER reduction. Moreover, the disruptive effect of *C. sakazakii* on the monolayers was confirmed by an increase in the permeability of monolayers to FITC-dextran and this change could be reversed by YL20 treatment, indicating that YL20 could play an important role in protecting intestinal epithelial barrier integrity. This was consistent with several previous studies. Ruchi Jariwala et al. reported that all strains, including the nine lactobacillus strains (*Limosilactobacillus fermentum* FA-1,FA-5, GKI-1, GPI-3, GPI-7, and IIs11.2; *Lactobacillus helveticus* FA-7; *Lactiplantibacillus plantarum* GRI-2; *L. salivarius* GPI-1(S)) and LGG, were able to reverse enteropathogenic *E. coli*-induced TEER decreases and permeability increases across Caco-2 monolayers [47]. Fan et al. also determined that *C. sakazakii* infection induced a reduction in the TEER of Caco-2 monolayers in a time-dependent manner, and this TEER reduction was alleviated by *B. fragilis* ZY-312 treatment [5].

The research of probiotics against *C. sakazakiiis*-induced NEC in vivo is limited as compared to other enteropathogens. *Lactobacillus lactis* has been confirmed to reduce NEC incidence and severity in a preterm rabbit model [14]. Conditioned medium from *B. infantis* was demonstrated to mitigate *C. sakazakii*-induced NEC in newborn mice by preventing the reduction of body weight, the apoptosis of enterocytes, and the reduction of mucin production [15]. Here, our data confirmed that YL20 administration could increase body weight, reduce mortality and intestinal epithelium damage in *C. sakazakii*-infected mice. Additionally, YL20 could also restore the number of goblet cells, the production of MUC2, and the expression of ZO-1, indicating its protective effect in intestinal barrier function against pathogen infection. Furthermore, YL20 treatment alone could also increase villus height and *Lgr5* mRNA level as well as up-regulate the number of goblet cells and the levels of MUC2. Combined with the results of organoid experiment in vitro, YL20 could contribute to intestine development and intestinal mucosal barrier formation by promoting ISC-mediated epithelial cell proliferation.

Ariadnna et al. demonstrated that flagella produced by *Cronobacter* species could activate pro-inflammatory cytokines TNF-α and IL-8 in macrophage derivatives from human monocytes [48]. *C. sakazakii* infection could up-regulate the relative mRNA levels of COX-2, IL-1β, IL-6, IL-8, iNOS, and TNF-α in human brain microvascular endothelial cells [49]. In this study, *C. sakazakii* infection was shown to stimulate the activation of pro-inflammatory factors such as IL-1β, IL-6, and TNFα in mice, and these *C. sakazakii*-induced inflammatory responses could be mitigated by YL20 administration. This agreed well with a previous report that *B. fragilis* ZY-312 could attenuate clinical symptoms and decrease the levels of TNF-α and IFN-γ in *C. sakazakii*-infected neonatal rats [5].

At present, there are few reports about the effect of *C. sakazakii* on intestinal microbiota. Recently, Alfreda et al. investigated the inhibitory effect of a potential synbiotic (six lactic acid bacteria strains and Vivinal GOS) on the growth of *C. sakazakii*, using a simulator of the human intestinal microbial ecosystem (SHIME) inoculated with infant fecal matter [50]. They reported a strong negative correlation between *Cronobacter* and *Bifidobacterium*, suggesting that the presence of *Bifidobacterium* in the gut of healthy infants could contribute to the inhibition of *C. sakazakii*. Premature or low birth weight infants may be more susceptible to *C. sakazakii* infection because they may lack some beneficial gut microbiota, including *Bacteroides* and *bifidobacteria* [51,52,53]. Consistent with Alfreda’s reports, our data confirmed that *C. sakazakii* infection led to the decrease in the abundance of *Bifidobacterium*. *C. sakazakii* infection also reduced the populations of beneficial bacteria *Bacteroidetes* and *Lactobacillus*, and enhanced the potential enteropathogenic bacteria *Enterobacterium* and *Enterococcus*, while these changes were partly reversed by the administration of YL20.

In this study, *L. salivarius* YL20 treatment was demonstrated to restore the budding and growth of organoids, up-regulate the mRNA level of *Lgr5*, *Zo-1* and *Occludin*, and promote the production of MUC2 in *C. sakazakiiis*-infected intestinal organoids. Additionally, YL20 could inhibit *C. sakazakii* invasion and reverse the *C. sakazakii*-induced low protein expression of ZO-1 and occludin in HT-29 cells. Moreover, YL20 could prevent TEER decreases and corresponding permeability increases across *C. sakazakii*-infected Caco-2 monolayers. Furthermore, YL20 could effectively attenuate epithelial barrier damage and inflammation, thereby preventing the development of NEC in neonatal mice. Our findings have demonstrated for the first time that YL20 can improve intestinal stem cell function and enhance intestinal barrier integrity, thus contributing to the prevention of *C. sakazakii*-induced NEC.

## Figures and Tables

**Table 1:** Primers used in real-time PCR.

Gene Name	Primer Sequence
*Tnf-α*	GCTCTGTGAAGGGAATGGGTGTT GTCCAGGTCACTGTCCCAGCATC
*Il-6*	ACTTCCATCCAGTTGCCTTCTTG TGTTGGGAGTGGTATCCTCTGTG
*Il-1β*	CAGAGTTCCCCAACTGGTACATC GGGAAGGCATTAGAAACAGTCC
*Gapdh*	TGCCCAGAACATCATCCCT TCCTCAGTGTAGCCCAAG
*Muc2*	GACGGCGATGTCTACCGATT TCCTTGTAGGAGTCTCGGCA
*Lgr5*	CCTACTCGAAGACTTACCCAGT GCATTGGGGTGAATGATAGCA
*Zo-1*	GTTGGTACGGTGCCCTGAAAGA GCTGACAGGTAGGACAGACGAT
*Occludin*	TGGCAAGCGATCATACCCAGAG CTGCCTGAAGTCATCCACACTC

**Table 2:** Primers used for bacteriological analyses.

Target Organism	Oligonucleotide Sequence (5′–3′)	Reference
Total bacterial	F′-CGGTGAATACGTTCYCGG R′-GGWTACCTTGTTACGACTT	[24]
*Bacteroidetes*	F′-GGTGTCGGCTTAAGTGCCAT R′-CGGAYGTAAGGGCCGTGC	[25]
*Lactobacillus*	F′-AGCAGTAGGGAATCTTCCA R′-CACCGCTACACATGGAG	[26]
*Bifidobacterium*	F′-AGGGTTCGATTCTGGCTCAG R′-CATCCGGCATTACCACCC	[26]
*Enterobacteriaceae*	F′-CATTGACGTTACCCGCAGAAGAAG R′-CTCTACGAGACTCAAGCTTGC	[27]
*Enterococcus*	F′-CCCTTATTGTTAGTTGCCATCAT R′-ACTCGTTGTACTTCCCATTGT	[28]
*Clostridium perfringens*	F′-CGCATAACGTTGAAAGATGG R′-CCTTGGTAGGCCGTTACCC	[29]

**Table 3:** Antagonistic activity of lactic acid bacteria against *C. sakazakii*.

Bacterial Strain	Diameter (mm)
*Lacticaseibacillus salivarius* YL20	12.5 ^a^ ± 0.3
*Lactobacillus gasseri* GM20	12.1 ^a^ ± 0.8
*Lacticaseibacillus rhamnosus* SW01	11.1 ^a^ ± 0.7
*Lacticaseibacillus rhamnosus* GG	11.9 ^a^ ± 0.7

^a^ Means in the column with the same superscript letter are not significantly different.

**Table 4:** Antibiotic susceptibility of *L. salivarius* YL20.

Antibiotic	Content	Inhibition Zone Diameter (mm)	Sensitivity
Penicillin	10 IU	7.1 ± 0.2	R
Streptomycin	10 μg	10.3 ± 0.3	R
Gentamicin	10 μg	17.2 ± 0.6	S
kanamycin	30 μg	19.2 ± 0.8	S
Tetracycline	30 μg	35.5 ± 0.5	S
Chloramphenicol	30 μg	26.3 ± 0.8	S
Ciprofloxacin	5 μg	27.9 ± 0.9	S
Rifampicin	5 μg	32.5 ± 0.7	S
Cephalothi	30 μg	30.2 ± 0.8	S
Cefotaxime Sodium	30 μg	34.8 ± 0.6	S
Ceftazidime	30 μg	22.5 ± 0.7	S
Erythromycin	15 μg	24.0 ± 0.7	S
Vancomycin	30 μg	15.8 ± 0.8	S
Cotrimoxazole	1.25 μg	20.3 ± 0.8	S
Imipenem	10 μg	36.0 ± 0.8	S

S: susceptible, R: resistance.

**Table 5:** Effect of YL20 on the abundance of dominant groups of bacteria (Lg [copies/g]).

Target Organism	Control	CS	YL20	CS + YL20
Total bacterial	42.45 ± 0.33 ^a^	42.16 ± 0.20 ^a^	42.39 ± 0.38 ^a^	41.97 ± 0.19 ^a^
*Bacteroidetes*	4.98 ± 0.32 ^a^	3.95 ± 0.13 ^b^	4.22 ± 0.17 ^ab^	4.01 ± 0.14 ^ab^
*Lactobacillus*	7.17 ± 0.12 ^b^	5.87 ± 0.07 ^c^	8.32 ± 0.05 ^a^	7.33 ± 0.04 ^b^
*Bifidobacterium*	5.68 ± 0.12 ^a^	4.32 ± 0.13 ^b^	5.35 ± 0.25 ^ab^	5.13 ± 0.19 ^ab^
*Enterobacteriaceae*	5.20 ± 0.04 ^b^	6.30 ± 0.08 ^a^	4.28 ± 0.08 ^c^	5.33 ± 0.05 ^b^
*Enterococcus*	5.53 ± 0.11 ^b^	6.69 ± 0.23 ^a^	4.93 ± 0.15 ^c^	5.81 ± 0.32 ^ab^
*Clostridium perfringens*	7.10 ± 0.16 ^a^	7.31 ± 0.20 ^a^	6.93 ± 0.28 ^a^	7.45 ± 0.13 ^a^

^a, b, c^ Values with different superscripts within the same row indicate significant differences, *p* < 0.05.

## Data Availability

The data obtained in this study are available on request from the author.

## References

[1] Samuels N., van de Graaf R., Been J.V., de Jonge R.C., Hanff L.M., Wijnen R.M., Kornelisse R.F., Reiss I.K., Vermeulen M.J. (2016). Necrotising enterocolitis and mortality in preterm infants after introduction of probiotics: A quasi-experimental study. Sci. Rep..

[2] Battersby C., Santhalingam T., Costeloe K., Modi N. (2018). Incidence of neonatal necrotising enterocolitis in high-income countries: A systematic review. Arch. Dis. Child. Fetal Neonatal Ed..

[3] Hunter C.J., Podd B., Ford H.R., Camerini V. (2008). Evidence vs. experience in neonatal practices in necrotizing enterocolitis. J. Perinatol..

[4] Lin P.W., Nasr T.R., Stoll B.J. (2008). Necrotizing enterocolitis: Recent scientific advances in pathophysiology and prevention. Semin. Perinatol..

[5] Fan H., Chen Z., Lin R., Liu Y., Wu X., Puthiyakunnon S., Wang Y., Zhu B., Zhang Q., Bai Y. (2019). Bacteroides fragilis Strain ZY-312 Defense against Cronobacter sakazakii-Induced Necrotizing Enterocolitis In Vitro and in a Neonatal Rat Model. mSystems.

[6] Nanthakumar N.N., Fusunyan R.D., Sanderson I., Walker W.A. (2000). Inflammation in the developing human intestine: A possible pathophysiologic contribution to necrotizing enterocolitis. Proc. Natl. Acad. Sci. USA.

[7] Henry M., Fouladkhah A. (2019). Outbreak History, Biofilm Formation, and Preventive Measures for Control of *Cronobacter sakazakii* in Infant Formula and Infant Care Settings. Microorganisms.

[8] Bowen A.B., Braden C.R. (2006). Invasive Enterobacter sakazakii disease in infants. Emerg. Infect. Dis..

[9] Ke A., Parreira V.R., Goodridge L., Farber J.M. (2021). Current and Future Perspectives on the Role of Probiotics, Prebiotics, and Synbiotics in Controlling Pathogenic *Cronobacter* Spp. in Infants. Front. Microbiol..

[10] Holý O., Cruz-Córdova A., Xicohtencatl-Cortes J., Hochel I., Parra-Flores J., Petrželová J., Fačevicová K., Forsythe S., Alsonosi A. (2019). Occurrence of virulence factors in Cronobacter sakazakii and Cronobacter malonaticus originated from clinical samples. Microb. Pathog..

[11] Seghesio E., De Geyter C., Vandenplas Y. (2021). Probiotics in the Prevention and Treatment of Necrotizing Enterocolitis. Pediatr. Gastroenterol. Hepatol. Nutr..

[12] Lin H.C., Hsu C.H., Chen H.L., Chung M.Y., Hsu J.F., Lien R.I., Tsao L.Y., Chen C.H., Su B.H. (2008). Oral probiotics prevent necrotizing enterocolitis in very low birth weight preterm infants: A multicenter, randomized, controlled trial. Pediatrics.

[13] Braga T.D., da Silva G.A., de Lira P.I., de Carvalho Lima M. (2011). Efficacy of Bifidobacterium breve and Lactobacillus casei oral supplementation on necrotizing enterocolitis in very-low-birth-weight preterm infants: A double-blind, randomized, controlled trial. Am. J. Clin. Nutr..

[14] Gurien L.A., Stallings-Archer K., Smith S.D. (2018). Probiotic Lactococcus lactis decreases incidence and severity of necrotizing enterocolitis in a preterm animal model. J. Neonatal. Perinatal. Med..

[15] Weng M., Ganguli K., Zhu W., Shi H.N., Walker W.A. (2014). Conditioned medium from Bifidobacteria infantis protects against Cronobacter sakazakii-induced intestinal inflammation in newborn mice. Am. J. Physiol. Gastrointest. Liver Physiol..

[16] Williams J.M., Duckworth C.A., Burkitt M.D., Watson A.J., Campbell B.J., Pritchard D.M. (2015). Epithelial cell shedding and barrier function: A matter of life and death at the small intestinal villus tip. Vet. Pathol..

[17] Hollister E.B., Gao C., Versalovic J. (2014). Compositional and functional features of the gastrointestinal microbiome and their effects on human health. Gastroenterology.

[18] Sun J. (2017). Intestinal organoid as an in vitro model in studying host-microbial interactions. Front. Biol..

[19] Lee S.B., Han S.H., Park S. (2018). Long-Term Culture of Intestinal Organoids. Methods Mol. Biol..

[20] Hou Q., Ye L., Liu H., Huang L., Yang Q., Turner J.R., Yu Q. (2018). Lactobacillus accelerates ISCs regeneration to protect the integrity of intestinal mucosa through activation of STAT3 signaling pathway induced by LPLs secretion of IL-22. Cell Death Differ..

[21] Deng K., Chen T., Wu Q., Xin H., Wei Q., Hu P., Wang X., Wang X., Wei H., Shah N.P. (2015). In vitro and in vivo examination of anticolonization of pathogens by Lactobacillus paracasei FJ861111.1. J. Dairy Sci..

[22] Huang R., Tao X., Wan C., Li S., Xu H., Xu F., Shah N.P., Wei H. (2015). In vitro probiotic characteristics of Lactobacillus plantarum ZDY 2013 and its modulatory effect on gut microbiota of mice. J. Dairy Sci..

[23] Béduneau A., Tempesta C., Fimbel S., Pellequer Y., Jannin V., Demarne F., Lamprecht A. (2014). A tunable Caco-2/HT29-MTX co-culture model mimicking variable permeabilities of the human intestine obtained by an original seeding procedure. Eur. J. Pharm. Biopharm..

[24] Yang L., Bian G., Su Y., Zhu W. (2014). Comparison of faecal microbial community of lantang, bama, erhualian, meishan, xiaomeishan, duroc, landrace, and yorkshire sows. Asian-Australas. J. Anim. Sci..

[25] Kang M., Mischel R.A., Bhave S., Komla E., Cho A., Huang C., Dewey W.L., Akbarali H.I. (2017). The effect of gut microbiome on tolerance to morphine mediated antinociception in mice. Sci. Rep..

[26] Steed H., Macfarlane G.T., Blackett K.L., Macfarlane S., Miller M.H., Bahrami B., Dillon J.F. (2011). Bacterial translocation in cirrhosis is not caused by an abnormal small bowel gut microbiota. FEMS Immunol. Med. Microbiol..

[27] Bartosch S., Fite A., Macfarlane G.T., McMurdo M.E. (2004). Characterization of bacterial communities in feces from healthy elderly volunteers and hospitalized elderly patients by using real-time PCR and effects of antibiotic treatment on the fecal microbiota. Appl. Environ. Microbiol..

[28] Rinttilä T., Kassinen A., Malinen E., Krogius L., Palva A. (2004). Development of an extensive set of 16S rDNA-targeted primers for quantification of pathogenic and indigenous bacteria in faecal samples by real-time PCR. J. Appl. Microbiol..

[29] Wise M.G., Siragusa G.R. (2005). Quantitative detection of Clostridium perfringens in the broiler fowl gastrointestinal tract by real-time PCR. Appl. Environ. Microbiol..

[30] Giri C.P., Shima K., Tall B.D., Curtis S., Sathyamoorthy V., Hanisch B., Kim K.S., Kopecko D.J. (2012). Cronobacter spp. (previously Enterobacter sakazakii) invade and translocate across both cultured human intestinal epithelial cells and human brain microvascular endothelial cells. Microb. Pathog..

[31] Li A., Jiang X., Wang Y., Zhang L., Zhang H., Mehmood K., Li Z., Waqas M., Li J. (2019). The impact of Bacillus subtilis 18 isolated from Tibetan yaks on growth performance and gut microbial community in mice. Microb. Pathog..

[32] Kaiko G.E., Ryu S.H., Koues O.I., Collins P.L., Solnica-Krezel L., Pearce E.J., Pearce E.L., Oltz E.M., Stappenbeck T.S. (2016). The Colonic Crypt Protects Stem Cells from Microbiota-Derived Metabolites. Cell.

[33] Yan F., Liu L., Cao H., Moore D.J., Washington M.K., Wang B., Peek R.M., Acra S.A., Polk D.B. (2017). Neonatal colonization of mice with LGG promotes intestinal development and decreases susceptibility to colitis in adulthood. Mucosal Immunol..

[34] Aoki-Yoshida A., Saito S., Fukiya S., Aoki R., Takayama Y., Suzuki C., Sonoyama K. (2016). Lactobacillus rhamnosus GG increases Toll-like receptor 3 gene expression in murine small intestine ex vivo and in vivo. Benef. Microbes.

[35] Lee Y.S., Kim T.Y., Kim Y., Lee S.H., Kim S., Kang S.W., Yang J.Y., Baek I.J., Sung Y.H., Park Y.Y. (2018). Microbiota-Derived Lactate Accelerates Intestinal Stem-Cell-Mediated Epithelial Development. Cell Host Microbe.

[36] Sittipo P., Pham H.Q., Park C.E., Kang G.U., Zhi Y., Ji H.J., Jang A., Seo H.S., Shin J.H., Lee Y.K. (2020). Irradiation-Induced Intestinal Damage Is Recovered by the Indigenous Gut Bacteria *Lactobacillus acidophilus*. Front. Cell. Infect. Microbiol..

[37] Mack D.R., Michail S., Wei S., McDougall L., Hollingsworth M.A. (1999). Probiotics inhibit enteropathogenic E. coli adherence in vitro by inducing intestinal mucin gene expression. Am. J. Physiol..

[38] Mack D.R., Ahrne S., Hyde L., Wei S., Hollingsworth M.A. (2003). Extracellular MUC3 mucin secretion follows adherence of Lactobacillus strains to intestinal epithelial cells in vitro. Gut.

[39] Lutgendorff F., Akkermans L.M., Söderholm J.D. (2008). The role of microbiota and probiotics in stress-induced gastro-intestinal damage. Curr. Mol. Med..

[40] Paone P., Cani P.D. (2020). Mucus barrier, mucins and gut microbiota: The expected slimy partners?. Gut.

[41] Ahl D., Liu H., Schreiber O., Roos S., Phillipson M., Holm L. (2016). Lactobacillus reuteri increases mucus thickness and ameliorates dextran sulphate sodium-induced colitis in mice. Acta Physiol. (Oxf.).

[42] González-Mariscal L., Betanzos A., Nava P., Jaramillo B.E. (2003). Tight junction proteins. Prog. Biophys. Mol. Biol..

[43] Qiao J., Sun Z., Liang D., Li H. (2020). *Lactobacillus salivarius* alleviates inflammation via NF-κB signaling in ETEC K88-induced IPEC-J2 cells. J. Anim. Sci. Biotechnol..

[44] Mange J.P., Stephan R., Borel N., Wild P., Kim K.S., Pospischil A., Lehner A. (2006). Adhesive properties of Enterobacter sakazakii to human epithelial and brain microvascular endothelial cells. BMC Microbiol..

[45] Campana R., van Hemert S., Baffone W. (2017). Strain-specific probiotic properties of lactic acid bacteria and their interference with human intestinal pathogens invasion. Gut Pathog..

[46] Collado M.C., Isolauri E., Salminen S. (2008). Specific probiotic strains and their combinations counteract adhesion of Enterobacter sakazakii to intestinal mucus. FEMS Microbiol. Lett..

[47] Jariwala R., Mandal H., Bagchi T. (2017). Indigenous lactobacilli strains of food and human sources reverse enteropathogenic E. coli O26:H11-induced damage in intestinal epithelial cell lines: Effect on redistribution of tight junction proteins. Microbiology (Reading).

[48] Cruz-Córdova A., Rocha-Ramírez L.M., Ochoa S.A., González-Pedrajo B., Espinosa N., Eslava C., Hernández-Chiñas U., Mendoza-Hernández G., Rodríguez-Leviz A., Valencia-Mayoral P. (2012). Flagella from five Cronobacter species induce pro-inflammatory cytokines in macrophage derivatives from human monocytes. PLoS ONE.

[49] Jin T., Guan N., Du Y., Zhang X., Li J., Xia X. (2021). Cronobacter sakazakii ATCC 29544 Translocated Human Brain Microvascular Endothelial Cells via Endocytosis, Apoptosis Induction, and Disruption of Tight Junction. Front. Microbiol..

[50] Ke A., Parreira V.R., Farber J.M., Goodridge L. (2022). Inhibition of Cronobacter sakazakii in an infant simulator of the human intestinal microbial ecosystem using a potential synbiotic. Front. Microbiol..

[51] Bäckhed F., Roswall J., Peng Y., Feng Q., Jia H., Kovatcheva-Datchary P., Li Y., Xia Y., Xie H., Zhong H. (2015). Dynamics and stabilization of the human gut microbiome during the first year of life. Cell Host Microbe.

[52] Yang I., Corwin E.J., Brennan P.A., Jordan S., Murphy J.R., Dunlop A. (2016). The infant microbiome: Implications for infant health and neurocognitive development. Nurs. Res..

[53] Cukrowska B., Bierła J.B., Zakrzewska M., Klukowski M., Maciorkowska E. (2020). The relationship between the infant gut microbiota and allergy. The role of Bifidobacterium breve and prebiotic oligosaccharides in the activation of anti-allergic mechanisms in early life. Nutrients.

